# Rogue waves lead to the instability in GaN semiconductors

**DOI:** 10.1038/srep12245

**Published:** 2015-07-24

**Authors:** M. E. Yahia, R. E. Tolba, N. A. El-Bedwehy, S. K. El-Labany, W. M. Moslem

**Affiliations:** 1 Faculty of Engineering and Natural Sciences, International University of Sarajevo (IUS), 71210, Ilidža, Sarajevo, Bosnia and Herzegovina; 2 Center for Theoretical Physics, The British University in Egypt (BUE), El-Shorouk City, Cairo 11837, Egypt; 3 Department of Mathematics, Faculty of Science, Damietta University, New Damietta, 34517, Egypt; 4 Department of Physics, Faculty of Science, Damietta University, New Damietta 34517, Egypt; 5 Department of Physics, Faculty of Science, Port Said University, Port Said 42521, Egypt

## Abstract

A new approach to understand the electron/hole interfaced plasma in GaN high electron mobility transistors (HEMTs). A quantum hydrodynamic model is constructed to include electrons/holes degenerate pressure, Bohm potential, and the exchange/correlation effect and then reduced to the nonlinear Schrödinger equation (NLSE). Numerical analysis of the latter predicts the rough (in)stability domains, which allow for the rogue waves to occur. Our results might give physical solution rather than the engineering one to the intrinsic problems in these high frequency/power transistors.

GaN physical characteristics have made it the ideal nominee for the high power, high temperature, and high frequency electronics applications^1^. GaN High Electron Mobility Transistors (HEMTs) is one of the most important examples application of GaN, which is characterized by very low noise figure because of the nature of the two dimensional electron gas 2DEG and the fact that there are less electron collisions. As a result of their noise performance they are increasingly in demand in low noise small signal wireless communications (mobile phones, satellite communications, TV broadcasting, broadband wireless internet connection, transmitter base station amplifiers), military applications (radars, missile seekers), voltage converters, microwave radio frequency power amplifiers, high-voltage switching, microwave sources, and radio astronomy^2^,^3^. However, great concerns about the reliability of GaN HEMTs-based power electronic devices have been raised due to self-heating which severely degrades their performance and lifetime^4^. The transient drain current collapse and gate-lag are two more ruinous accompanying mechanisms that are theorized to have occurred because of trapping^5^,^6^. Although, many researchers overwhelmingly combine on the location of the GaN HEMT traps, but the strange thing is that, they always give fuzzy reasons basically depend on random localization distribution of causes like impurities, lattice defect, and dislocations. Besides, they often give an inaccurate depiction of the quantum tunneling effect.

Recent research shed the light on many anomalous phenomena that have been observed in a variety of systems, ranging from discrete lattices^7^,^8^, fluids^9^,^10^, plasmas^11^, ultracold quantum gases^12^,^13^, optics^14^,^15^,^16^,^17^, lasers^18^,^19^,^20^, optical fibers^21^,^22^,^23^. Their similar characteristics that are governed by the nonlinear Schrödinger equation (NLSE) solutions could be considered as the clue to understand their nature. Rogue waves are one of the most distinguished embodiments of extreme events in nonlinear dynamics, which are attributed to the modulation instability (MI). The MI or sideband instability is one of the most pervasive types of instabilities in nature^24^, it originates as an interaction between strong carrier harmonic waves and small sidebands. In this aspect, the MI plays a great role as an “intrinsic noise” in triggering a self-organization mechanism^25^. Instabilities and spontaneous pattern formation in numerous natural and artificial systems caused by rogue waves can have catastrophic consequences, but the situation could be better if we know their multiple scale hierarchies.

Quantum effects are also expected to play an important role in GaN HEMT under the conditions of extreme events. This situation becomes clear when the energy density of the plasma oscillations is equal or comparable to the Fermi electron thermal energy density. Even though the electron density is almost low in some applications for considering the typical quantum mechanical effects, such as tunneling, the situation in other applications may be supposed to be of quantum nature. In this report, we are particularly interested in the theoretical description of the steady state instability with a direct current for ungated two-dimensional electron-hole plasma in the GaN HEMT. We are using quantum plasma hydrodynamics approach, which is characterized by small wave numbers and valid when the mean-free path for electron-electron collisions is smaller than both the device length and the mean-free path for collisions with impurities and phonons^26^. However, it gives qualitatively correct results even if those conditions are not fulfilled^27^,^28^.

The quantum fluid model is derived from the Wigner—Poisson system. It would be interesting to introduce different quantum effects, to examine the MI and to test the reaction of the quantum effects on the rogue wave profile. For this purpose, we focus our attention on the specific scenario of balancing between the group dispersion and the nonlinear effects caused by pressure force, Bohm and exchange-correlation potentials. It would be motivating to assess in more detail the spectra of an unstable mode zone, and it would be promising to benchmark our results with an experimental observation. Accordingly, the goal of our investigation is twofold: (i) to demonstrate the existence region of the acoustic rogue waves (ARWs) and (ii) examine how the ARWs are influenced by different quantum effects.

We consider the propagation of nonlinear electrostatic acoustic waves in a two-component collisionless plasma composed of electrons and holes. The nonlinear dynamics of such disturbances is governed by the normalized fluid equations for electrons


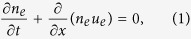



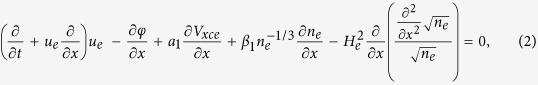


and for holes


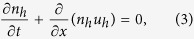



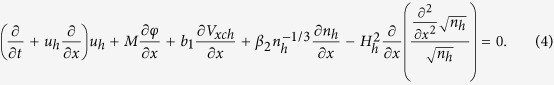


The system is closed by the Poisson equation


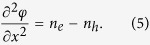


Here, *n*_*e*_ (*u*_*e*_) and *n*_*h*_ (*u*_*h*_) stand for the density (velocity) of the electrons and holes, respectively, *V*_*xce*,*h*_ are the electrons and holes exchange-correlation potentials, and *φ* is the electrostatic potential. It is interesting to clarify how the basic set of fluid equations describing the propagation of nonlinear acoustic solitons is obtained. Let us start with the velocity moment of the Boltzmann equation^29^


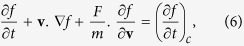


where *f* is the velocity distribution function, *F* is the force acting on the particles, and (∂*f*/∂*t*)_*c*_ is the time rate of change of *f* due to collisions. The continuity equations (1) and (3) are obtained by the lowest moment of Eq. (6), while the next moment of the Boltzmann equation is obtained by multiplying Eq. (6) by *mv* and integrating over *dv*. Then, we finally arrive at the momentum equations (2) and (4). Notice that the total force of the charged particles could be electric force, magnetic force, Bohm potential force (recoile force), exchange-correlation force, and pressure force. A detailed mathematical treatment of this issue can be found in Ref. [30].

The variables appearing in Eqs (1, 2, 3, 4, 5) have been appropriately normalized. Thus, *n*_*e*_ and *n*_*h*_ are normalized by the unperturbed electron (hole) number density *n*_*e*0_(*n*_*h*0_), *u*_*e*_ and *u*_*h*_ are normalized by the Fermi electron speed 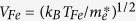 in terms of Fermi temperature *T*_*Fe*_, and *φ* is normalized by *k*_*B*_*T*_*Fe*_/*e*. The space and time variables are in units of the Fermi electron Debye radius *λ*_*DFe*_ = (*k*_*B*_*T*_*Fe*_*ε*_0_/*e*^2^*n*_*e*0_)^1/2^ and the inverse of the plasma frequency 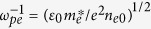, respectively, 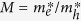 is the electron-to-hole effective mass ratio, *ε*_0_ is the permitivity, *e* is the magnitude of the electron charge, and *k*_*B*_ is the Boltzmann constant. Furthermore, *a*_1_ = (*K*_*B*_*T*_*Fe*_)^−1^, *b*_1_ = *M*(*K*_*B*_*T*_*Fh*_)^−1^, 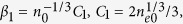 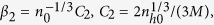 *H*_*e*_ = (*ħω*_*pe*_/2*K*_*B*_*T*_*Fe*_), *H*_*h*_ = (*ħω*_*pe*_/2*K*_*B*_*T*_*Fe*_)*M*. The non-relativistic pressure law^31^ of the degenerate electrons/holes is used in Eqs (2) and (4), where 
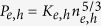
, 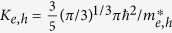, *ħ* is the Planck constant divided by 2*π*. The exchange-correlation potentials for the electrons and holes are given by 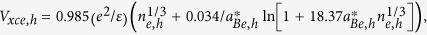 where 
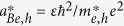
 and *ε* is the dielectric constant of the material.

To examine the dynamics of the one-dimensional ARWs propagating in an electron-hole plasma, we analyze the outgoing solutions of Eqs (1, 2, 3, 4, 5) by introducing the stretched coordinates^32^,^33^


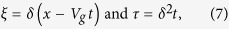


where *δ* is a small (real) parameter and *V*_*g*_ the envelope group velocity. The dependent variables are expanded as


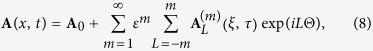


where


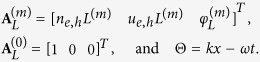


Here, *k* and *ω* are real variables representing the fundamental (carrier) wavenumber and frequency, respectively. Since 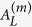 must be real, the coefficients in Eq. (8) have to satisfy the condition 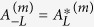, where the asterisk indicates the complex conjugate. Substituting (7) and (8) into Eqs (1, 2, 3, 4, 5) and collecting terms of the same powers of *δ* and different *m* and *L*, we obtain a system of equations where the compatibility condition yields the NLSE


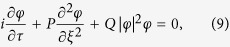


where *P* and *Q* are given in Ref. [32]. A detailed description of the mathematical method to obtain the NLSE can be found in Refs 32,33.

The NLSE (9) has a rational solution that is located in a nonzero background and localized both in the *ξ* and *τ* directions^33^,^34^ as


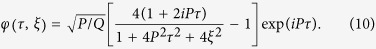


Solution (10) prognosticates the concentration of the ARWs energy into a small region that is caused by the nonlinear behavior of the plasma medium. Indeed, rogue waves are usually an envelope of a carrier wave with a wavelength smaller than the central region of the envelope. Rogue waves are able to focus (self-trapping) significant amounts of wave energy into a relatively small area, which may provide the basis for the illustration of the ARWs in semiconductor plasma.

It is interesting to mention that the NLSE (9) is a 1 + 1 dimensional evolution equation, which cannot fully describe the real physics of a multidimensional semiconductor device. However, due to the complexity of the used mathematical method it is not possible to derive the 2 + 1 dimensional eqution (9). Of course, the rogue waves that exist in higher dimensions are affected by the extra dimensions. To the best of our knowledge, the multi-dimensional NLSE cannot be derived except for the case of the strong magnetic field. The latter, causes the fluid gyromotion to be treated as a higher order effect. So, it will be interesting to consider a weakly three-dimensional modulation of the envelope solitons, i.e. when the wave propagates in one direction with weak transverse perturbations in the other two directions, and hence the transverse velocity components also appear at higher orders with respect to the parallel velocity component (see e.g.^35^,^36^).

The analyses of the NLSE (9) disclose the existence of either unstable or stable pulses. The appropriate tool to examine the region of (in)stabitliy of the envelope pulse amplitude for external perturbations is the sign of the ratio *P*/*Q*. For a negative *P*/*Q*, the modulated envelope is stable, while for a positive *P*/*Q* the modulated envelope will be unstable against external perturbations. In the latter case, the carrier wave is modulationally unstable; so it may either “collapse”, due to (possibly random) external perturbations, or lead to the formation of “bright” envelope modulated wavepackets, i.e. localized envelope “pulses” confining the carrier wave. The instability is usually saturated by the formation of a train of envelope pulses, the so-called bright solitons. These bright solitons can be stationary in time, but the system can also oscillate periodically back and forth between the soliton state and an almost homogeneous state, usually referred to as the Fermi-Pasta-Ulam oscillation^31^. For a negative *P*/*Q*, the carrier wave is modulationally “stable”, and dark solitons exist. Our aim is to study the case of an unstable solution for *P*/*Q* > 0, which is a typical situation for ARWs formations^37^.

In what follows, we analyze the parametric dependence of the rogue wave profile on the plasma density and wave number for GaN semiconductor bulk plasmas with typical parameters^38^,^39^,^40^ *n*_0_ ~ 10^22^ − 10^26^ *m*^−3^, 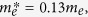 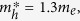 and *ε* = 11.3. It is worthwhile to first clear why our basic Eqs (2) and (4) are missing the damping terms as in Ref. [27]. Indeed, considering the collisions between electron-phonon (hole-phonon) is interesting and may lead to a wave collapse that will have a crucial effect on the instability of the quantum acoustic rogue wave. However, considering this effect is important when the time scale of the propagating wave is of the order of the collision time scale. Otherwise, if the collision time scale is too long compared to the wave time scale, then the wave initiates and propagates before the beginning of the collsions. For GaN material, the electron plasma period is of the order of a picosecond. The electron-ion (phonon) collision is given by *ν*_*ei*_ = *n*_0_*e*^2^*η*/*m*_*e*_, where *η* is the spesific resistivity^41^. Given the GaN parameters the collision period is of the order of a millisecond - microsecond. So, we can safely ignore the collision frequency in comparision to the plasma frequency and consider the plasma as collisionless.

First, the sign of the ratio *P*/*Q* is depicted in Fig. 1. The yellow region stands for *P*/*Q* > 0 and the white region stands for *P*/*Q* < 0. Figure 1a shows the positive region of *P*/*Q* for small wave numbers band (i.e. *k* = 0 − 7), which shows that there are three regions of instability. Increasing the wave number (*k* = 7 − 200) as depicted in Fig. 1b, it is obvious that if there is one cut-off region of instability (i.e. *k* ~ 40 − 100) then the instability region continuously exists. Of course, the *n*_*e*0_ − *k* plan is an important tool to precisely define the propagation region of the rogue waves. Now, we know the expected region and parameters that may allow the existence of rogue waves in a GaN semiconductor. However, could the quantum effects such as Bohm potential or exchange-correlation terms change the *n*_*e*0_ − *k* plan. In an analogous manner, we neglect/cancel the Bohm potential term (i.e. by putting *H*_*e*_ = *H*_*h*_ = 0) and redrew the *n*_*e*0_ − *k* graph as depicted in Fig. 1c. It is clear that there are only two narrow regions of instability at a low wave number band (i.e. *k* = 1 − 2). Furthermore, neglecting the exchange-correlation terms have no significant effect on the *n*_*e*0_ − *k* plan, so we did not include that figure here. Therefore, the presence of Bohm potential is the main quantum factor for creating instability in the GaN semiconductor.

It would be interesting to notice that the rogue semiconductor waves can be generated within the unstable zone that is represented by the yellow part of the *n*_*e*0 _− *k* plan. Creating a rogue wave as a self-trapping effect is due to sucking the energy/carriers from the background (as it depletes the 2-dimension electron gas (2DEG)) into a small area, which produces highly localized self-modulated pulses. The confinement of this energy/carriers cannot be produced without the tunneling effect of carriers (cf. Fig. 1c). The tunneling effect gives the electrons the way to penetrate the rogue wave trapping center, which will be near to the denser electron streams. This mechanism degrades pinch-off characteristics and decreases the GaN transconductance. These “bulk traps” in the GaN buffer region have been suggested to be the reason for dispersion in a GaN HEMTs, current collapse, and gate-lag^42^. Therefore, the relaxation time in current lag effects in HEMTs is increased.

Now, our aim is to numerically analyze the maximum ARWs envelope amplitude |*φ*_*M*_| for the GaN and investigate how the plasma number density *n*_*e*0_ and the wave number *k* alter its profile. In order to gain some insight, we have depicted the rogue wave profile as well as the contour plot of |*φ*_*M*_| with *n*_*e*0_ and *k* as in Fig. 2. The rogue wave profile that may propagate for GaN plasma parameters within the yellow or unstable region is depicted in Fig. 2a. We have divided the instability regions into three parts A, B, and C (cf. Fig. 1a,b). Figure 2a shows the region A, it is seen that an increase of the plasma number density would lead to the reduction of the rogue wave amplitude. However, for 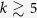 the wave amplitude has higher values than for *k* < 5. The same behavior is found in part B and is represented in Fig. 2b. The value of the maximum ARWs envelope amplitude |*φ*_*M*_| is much greater than (even a hundred times) Fig. 2a. It means that for larger wave numbers the rogue waves suck more energy, which causes high amplitude pulses. This analysis could give rise to the self-heating mechanism, where this localized envelope attracts and compresses more charged carriers energies in a very small bulk size. Then, the higher the carrier density, the more the temperature increases. Figure 2c corresponds to part C, it is clear that for a high plasma number density *n*_*e*0_ the maximum envelope amplitude decreases, but for a higher wave number the amplitude increases up to very high values.

The rogue waves depend on the carrier density and its profile is represented in Fig. 3. For example, its full wave growth-time (*ω*^−1^ ~ *t*) can be approximately calculated for *k* = 15 and *n*_*e*0_ ~ 10^25^ *m*^−3^ to be ~10 fs, which corresponds to the tranisent collapse in the carrier density. Finally, it is important to assign the collapse of the drain current to the rogue waves that are considered as attractors to self-trap the carrier’s energy into the GaN bulk. We think that, the self-heating region is produced gradually as the tunneling electrons exchange their energy with the lattice. This causes an increase of the resistance in the GaN edge (2DEG region). In order to avoid the consequences of the rogue wave trapping, one should manage the operation of the GaN in the stable zones.

In conclusion, we have shown that rogue waves can be exclusively responsible for the production and concentration of very high wave energy in the GaN semiconductor and its consequences related to the operation of the HEMTs. Analysis of the NLSE provides us with the existence regions for the rogue waves. The analysis is also able to qualitatively explain the transient collapse of the transconductance and the self-heating of the GaN.

## Additional Information

**How to cite this article**: Yahia, M. E. *et al.* Rogue waves lead to the instability in GaN semiconductors. *Sci. Rep.* **5**, 12245; doi: 10.1038/srep12245 (2015).

## Figures and Tables

**Figure 1:**
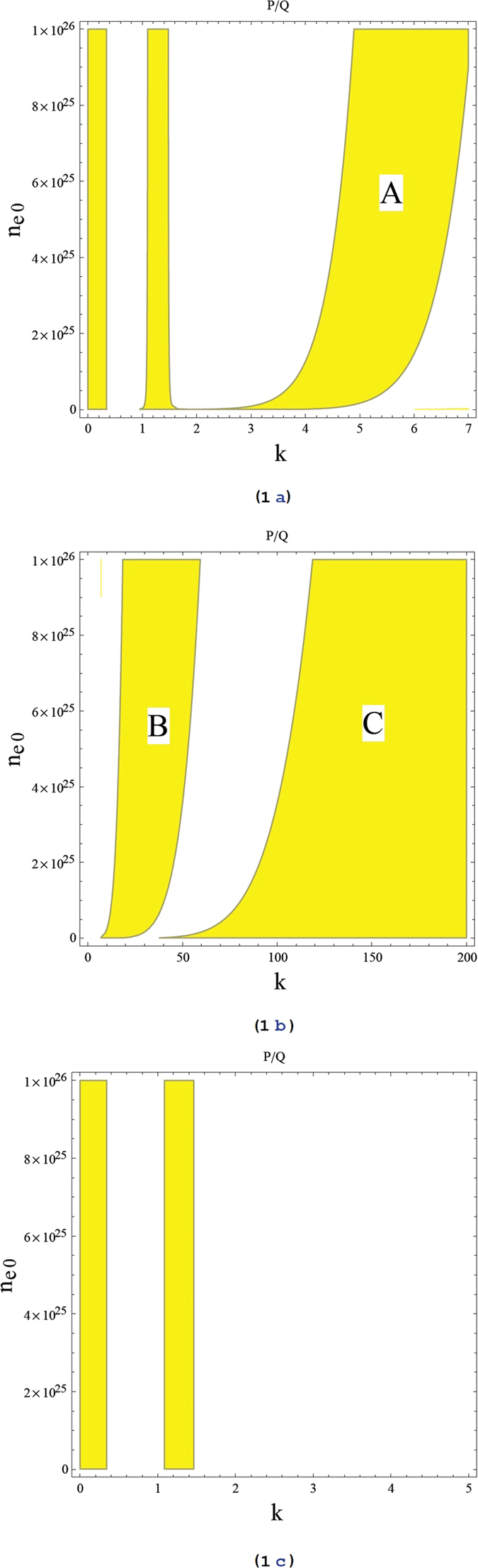
The ratio *P*/*Q* contour is depicted against the wave number *k* and electron number density *n*_*e*0_; the yellow (white) color represents the region where the unstable (stable) waves set in. (**a**) and (**b**) with all quantum effects and (**c**) without Bohm potential effect.

**Figure 2:**
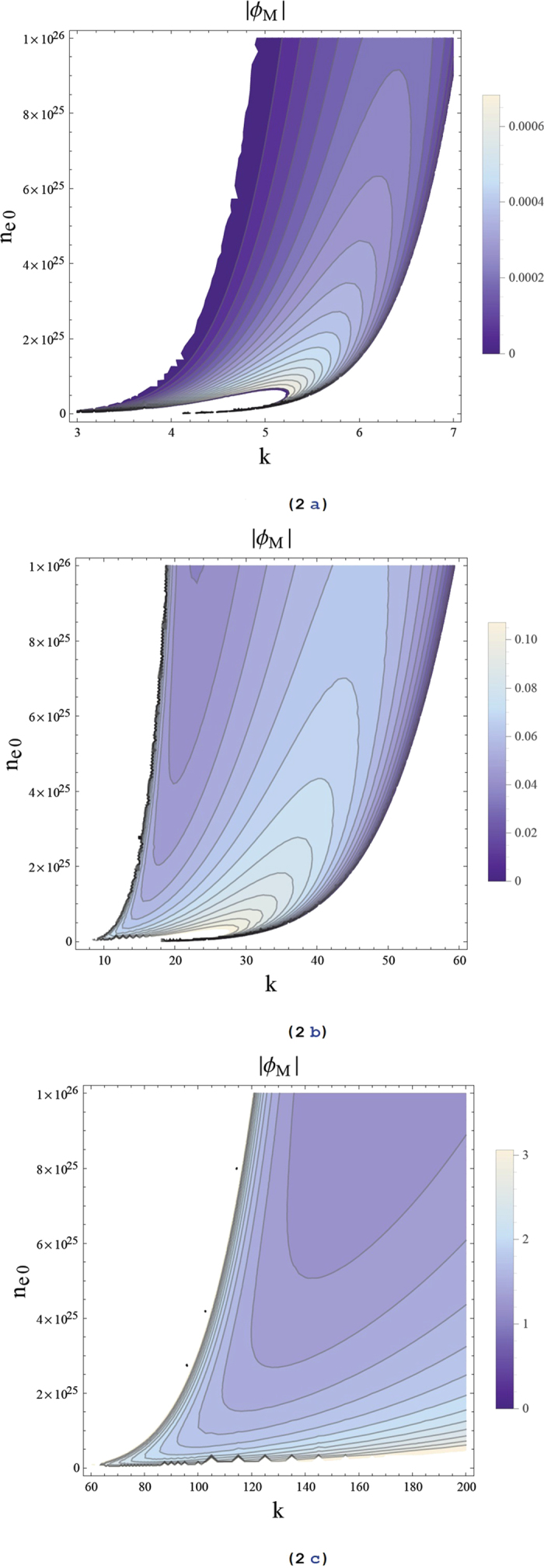
Maximum rogue wave amplitude |*φ*_*M*_| contour is depicted against the wave number *k* and electron number density *n*_*e*0_ for different regions of *k*. Light-colored regions correspond to high values of |*φ*_*M*_|.

**Figure 3:**
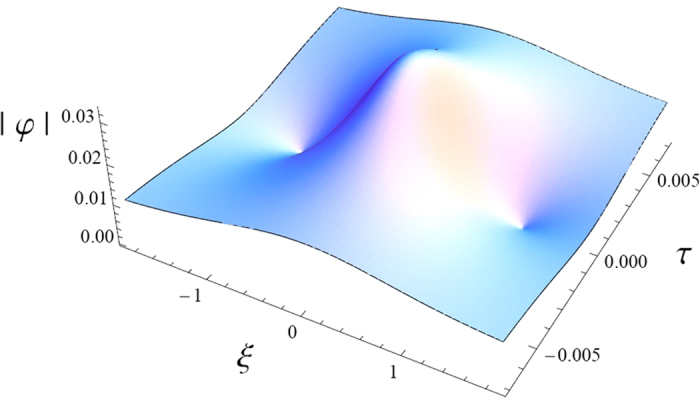
The rogue wave profile for carrier density *n*_*e*0_ = 10^25^ m^−3^ and *k* = 15.
